# The Effect of Latent Hepatitis B Infection on Autoimmune Hepatitis Treatment Outcomes

**DOI:** 10.7759/cureus.88997

**Published:** 2025-07-29

**Authors:** Sami Bahcebası, Nuh Mehmet Büyükberber, Osman Saglam

**Affiliations:** 1 Internal Medicine, Kayseri City Training and Research Hospital, Kayseri, TUR; 2 Gastroenterology and Hepatology, Kayseri City Hospital, Kayseri, TUR; 3 Gastroenterology and Hepatology, Afyonkarahisar Public Hospital, Afyonkarahisar, TUR

**Keywords:** anti-hbc igg, autoimmune hepatitis, latent hepatitis b, prophylaxis, treatment success

## Abstract

This study aimed to determine how latent hepatitis B virus (HBV) infection affects the treatment response and disease progression in patients with autoimmune hepatitis (AIH). We retrospectively analyzed clinical and laboratory data from 36 hepatitis B surface antigen (HBsAg)-negative AIH patients, classified based on anti-HBc immunoglobulin G (IgG) status to identify latent HBV presence. Among these, 13 patients (36.1%) were anti-HBc IgG-positive, indicating latent HBV infection, while 23 patients (63.9%) were negative. Liver function markers were assessed at diagnosis and after one year of standard immunosuppressive therapy to determine treatment response. Our results indicate that patients harboring latent HBV exhibit a poorer biochemical response to AIH treatment, with persistently elevated liver enzymes and impaired liver function markers compared to those without latent infection. These findings highlight the potential impact of latent HBV on disease progression and treatment efficacy in AIH. We therefore recommend comprehensive screening for latent HBV in all AIH patients before immunosuppressive therapy and consideration of antiviral prophylaxis to improve therapeutic outcomes.

## Introduction

Autoimmune hepatitis (AIH) is a chronic inflammatory liver disease of unknown etiology that can progress to cirrhosis and ultimately liver failure if left untreated. Patients may present with acute hepatitis or remain asymptomatic. While AIH is more common in women, it can affect individuals of all ages and genders. The inflammation results from an abnormal immune response to autoantigens in the liver, which contributes to its pathogenesis. Diagnosis is based on specific autoantibody positivity, elevated immunoglobulin G (IgG) levels, and characteristic histological findings on liver biopsy. These commonly include interface hepatitis and periportal necrosis, but may also involve lobular hepatitis, plasma cell infiltration, hepatocyte rosette formation, bile duct injury, and varying degrees of fibrosis.

Autoantibodies to be investigated in AIH include antinuclear antibody (ANA), anti-smooth muscle antibody (ASMA), liver-kidney microsomal type 1 antibody (LKM-1), and antimitochondrial antibody (AMA). ASMA positivity is commonly associated with type 1 AIH, while LKM-1 positivity is typically linked to type 2 AIH. However, it is important to note that both ASMA and LKM-1 can be negative in some cases, and other autoantibodies, such as ANA and SMA (smooth muscle antibodies), may also be present in type 1 AIH. Additionally, LC-1 positivity can occasionally be seen in Type 2 AIH. Occasionally, overlap syndromes with primary biliary cholangitis (PBC) or primary sclerosing cholangitis (PSC) occur, complicating the differential diagnosis. The treatment primarily involves corticosteroids and azathioprine to suppress abnormal immune responses. Ursodeoxycholic acid is added to the regimen if an overlap syndrome is suspected [1].

The Fibrosis-4 (Fib-4) score is a noninvasive tool used to assess liver fibrosis in AIH. A Fib-4 score >3.25 has 97% specificity and a positive predictive value of 65% for advanced fibrosis, whereas a score <1.45 has a 90% negative predictive value for advanced fibrosis [2]. Anti-HBc IgG positivity indicates previous exposure to the hepatitis B virus (HBV). It may signify chronic hepatitis B in cases with an active immune response or latent hepatitis B infection [3].

For patients undergoing immunosuppressive biological treatments, screening for latent hepatitis B is recommended, and prophylactic antiviral treatment should be administered if detected [4]. However, antiviral prophylaxis is not routinely recommended for patients with AIH receiving immunosuppressive agents. Nevertheless, latent hepatitis B infection may contribute to AIH pathogenesis. Occult hepatitis B has been observed in patients with high anti-HBc IgG positivity [5]. Although HBV-DNA is often negative in peripheral blood in these patients, HBV presence in liver tissue has been confirmed by biopsy [6,7]. Therefore, anti-HBc IgG testing at the time of AIH diagnosis is recommended for diagnosing occult hepatitis B [8]. In this study, we aimed to compare treatment response rates between patients with and without latent hepatitis B infection based on anti-HBc IgG results.

## Materials and methods

We employed a retrospective, single-center study design. We retrospectively reviewed the records of patients diagnosed with AIH at the Gastroenterology Clinic from January 1, 2019, to October 31, 2023. The study was conducted in accordance with the principles of the Declaration of Helsinki. All patients were HBsAg-negative. Patients were classified into two groups based on their Anti-HBc IgG status: Anti-HBc IgG-positive (n=13) and Anti-HBc IgG-negative (n=23). Liver function tests at diagnosis and one year after treatment were compared.

Aspartate aminotransferase (AST), alanine aminotransferase (ALT), gamma-glutamyl transferase (GGT), total bilirubin (T. bilirubin), direct bilirubin (D. bilirubin), albumin, international normalized ratio (INR), AST to platelet ratio index (APRI), and Fibrosis-4 (Fib-4) scores were recorded at diagnosis and one year after treatment as liver function tests. Autoimmune markers necessary for diagnosis included ANA, ASMA, LKM-1, and AMA. IgG levels and pathological results were recorded. Hepatitis markers, including HBsAg, anti-HBs, anti-HBc IgG, anti-HCV, and anti-HIV antibodies, were documented. Patients without a one-year follow-up were excluded from the study.

The recommended cutoff values to assess normalization of liver function within the first year after treatment were >40 U/L for ALT and AST, >1.2 mg/dL for T. bilirubin, >0.3 mg/dL for D. bilirubin, and <40 g/L for albumin. In our study, the normalization of all cutoffs was considered and applied to all patients, ensuring consistency in the interpretation of the results. Based on these cutoff values, the number of patients who did not reach normal values was calculated. Response to treatment was evaluated according to criteria defined by the International Autoimmune Hepatitis Working Group [9].

Inclusion criteria

We included patients aged 18-65 with AIH but without known chronic liver disease.

Exclusion criteria

Patients under 18 years of age and over 65 years, pregnant women, and individuals with a known history of chronic liver disease were excluded from the study. Additionally, patients diagnosed with other conditions that impair liver function, those with toxic hepatitis or viral hepatitis, and AIH patients without at least one year of follow-up were also excluded.

Statistical analysis

Data were analyzed using SPSS Statistics version 22.0 (IBM Corp., Armonk, NY). The Shapiro-Wilk test was used to assess the normality of data distribution. Multivariate analysis was performed using MANOVA (multivariate analysis of variance) for parametric data, Kruskal-Wallis test for non-parametric and ordinal data, and Chi-square test for nominal data. For comparisons between independent groups, the t-test was used for parametric data and the Mann-Whitney U test for non-parametric data. For comparisons before and after treatment, a paired samples t-test was used for parametric data and a Wilcoxon signed-rank test for non-parametric data. Parametric data are expressed as mean ± standard deviation (SD), non-parametric and ordinal data as median (minimum-maximum), and nominal data as number (%). Statistical significance was set at p<0.05.

## Results

Our study included 36 patients: five males (13.9%) and 31 females (86.1%). All diagnoses were established through clinical, laboratory, and pathological evaluations. ANA positivity was observed in 26 patients (72.2%), AMA positivity in eight patients (22.2%), and ASMA positivity in 18 patients (50.0%). Anti-LKM-1 was not detected in any patient. Liver biopsy findings revealed AIH in 27 patients (75.0%), AIH with cirrhosis in one patient (2.8%), AIH with hepatosteatosis in one patient (2.8%), and AIH with PBC overlap syndrome in seven patients (19.4%).

Analysis of anti-HBc IgG total and treatment response in autoimmune hepatitis patients by biopsy categories

A cross-tabulation analysis was performed to evaluate the relationship between anti-HBc IgG status and biopsy categories (including cirrhosis, hepatitis, hepatitis with PBC, and AIH with steatosis) and treatment response. A total of 36 patients were included.

Among patients who were Anti-HBc IgG-negative, one (2.8%) had cirrhosis, 17 (47.2%) had hepatitis, and five (13.9%) had hepatitis with PBC, while none had AIH with steatosis. The patient with cirrhosis was included in the analysis because their liver biopsy at the time of diagnosis did not show chronic liver disease but rather acute findings. Therefore, according to the study's diagnostic criteria, the patient was eligible for inclusion despite the cirrhosis diagnosis in the biopsy. In contrast, among anti-HBc IgG-positive patients, 10 (27.8%) had hepatitis, two (5.6%) had hepatitis with PBC, and 1 (2.8%) had AIH with steatosis. No cases of cirrhosis were observed in this group. Statistical analysis using the Pearson Chi-square test revealed no significant association between anti-HBc IgG status and biopsy category distribution (χ² = 2.517, df = 3, p = 0.472), indicating that biopsy patterns were comparable between anti-HBc IgG-positive and negative patients.

In terms of treatment response, non-responders included four patients (11.1%) with hepatitis and two patients (5.6%) with hepatitis and PBC overlap; no patients in this group had cirrhosis or AIH with steatosis. Among responders, one patient (2.8%) had cirrhosis, 23 (63.9%) had hepatitis, five (13.9%) had hepatitis with PBC, and one (2.8%) had AIH with steatosis. The association between biopsy categories and treatment response was not statistically significant (Pearson χ² = 1.181, df = 3, p = 0.758), suggesting that histological presentation did not influence treatment outcome.

The findings suggest that neither anti-HBc IgG status nor treatment response significantly influences the distribution of biopsy categories in patients with AIH.

Additional autoimmune diseases

As for additional autoimmune diseases, one patient had primary hyperparathyroidism, two patients had systemic lupus erythematosus, and one patient had both systemic lupus erythematosus and primary hyperparathyroidism.

Treatment regimen

Patients were treated with methylprednisolone and azathioprine as recommended by guidelines. Prednisolone was started at a dose of 0.5 mg/kg body weight, and azathioprine was added within two weeks of steroid initiation. Steroid doses were reduced during follow-up, and maintenance treatment was continued with low-dose azathioprine (1 mg/kg body weight) and low-dose prednisone (5 mg/day).

After one year of treatment, 30 patients showed a clinical response, while six patients did not respond. Multivariate analysis was performed to evaluate parameters associated with non-response. No significant relationship was found between treatment unresponsiveness and age, sex, ANA, ASMA, WBC, lymphocyte, neutrophil, platelet count, AST, ALT, GGT, ALP, INR, total and direct bilirubin, albumin, IgG, Fib-4 score, or APRI index at diagnosis. However, a significant relationship was found between anti-HBsAg and anti-HBc IgG positivity and treatment unresponsiveness (Table 1).

**Table 1:** Multivariate analysis of factors associated with response to autoimmune hepatitis treatment The symbol t indicates results from the independent samples t-test, while U represents the Mann–Whitney U test and χ² denotes the Pearson Chi-Square test. A p-value less than 0.05 was considered statistically significant ALT: alanine aminotransferase; AMA: antimitochondrial antibody; ANA: antinuclear antibody; APRI: AST to platelet ratio index; ASMA: anti-smooth muscle antibody; AST: aspartate aminotransferas; Fib-4: Fibrosis-4; GGT: gamma-glutamyl transferase; IgG: immunoglobulin G; INR: international normalized ratio; SD: standard deviation; WBC: white blood cells

Parameter	Responders (n = 30)	Non-responders (n = 6)	P-value	Test statistic
Age, years, median (range)	50 (18–65)	54 (44–63)	0.233	U = 61.5
Gender, n (%)	Female: 25 (83.3%)	Female: 6 (100%)	0.281	χ² = 1.161
	Male: 5 (16.7%)	Male: 0 (0%)		
ANA, n (%)	Negative: 8 (26.7%)	Negative: 2 (33.3%)	0.739	χ² = 0.111
	Positive: 22 (73.3%)	Positive: 4 (66.7%)		
AMA, n (%)	Negative: 24 (80.0%)	Negative: 4 (66.7%)	0.473	χ² = 0.514
	Positive: 6 (20.0%)	Positive: 2 (33.3%)		
ASMA, n (%)	Negative: 15 (50.0%)	Negative: 3 (50.0%)	1	χ² = 0.000
	Positive: 15 (50.0%)	Positive: 3 (50.0%)		
Anti-HBc IgG, n (%)	Negative: 22 (73.3%)	Negative: 1 (16.7%)	0.008	χ² = 6.959
	Positive: 8 (26.7%)	Positive: 5 (83.3%)		
WBC, μL, mean ± SD	6684 ± 1599	6320 ± 2316	0.64	t = -0.472 (df = 34)
Lymphocyte, μL, mean ± SD	2106 ± 797	1803 ± 838	0.406	t = -0.842 (df = 34)
Albumin, g/L, mean ± SD	39.3 ± 5.7	38.6 ± 5.9	0.805	t = -0.249 (df = 34)
Knodel HAI, median (range)	8/18 (3–13)	8/18 (3–12)	0.798	U = 84
Ishak score, median (range)	4/6 (0–6)	4/6 (0–5)	0.486	U = 74
ALT, U/L, median (range)	262 (7–2020)	587 (28–1055)	0.467	U = 72
AST, U/L, median (range)	200 (16–1357)	592 (34–1224)	0.371	U = 68
AST/ALT ratio, median (range)	0.84 (0.44–1.76)	1.10 (0.98–1.63)	0.042	U = 42
Total bilirubin, mg/dL, median (range)	1.85 (0.2–19.7)	5.3 (0.5–13.1)	0.349	U = 67.5
Direct bilirubin, mg/dL, median (range)	0.7 (0.1–15.5)	4.0 (0.3–11.0)	0.186	U = 58
GGT, U/L, median (range)	152 (7–831)	326 (63–829)	0.103	U = 51
ALP, U/L, median (range)	145 (70–1058)	198 (107–371)	0.394	U = 69
INR, median (range)	1.11 (0.9–1.93)	1.14 (1.03–1.42)	0.634	U = 78.5
Neutrophil, μL, median (range)	3585 (2360–6350)	3040 (1870–7360)	0.349	U = 113
Platelet, 10³/μL, median (range)	266 (117–908)	197 (160–347)	0.217	U = 119.5
IgG, mg/dL, median (range)	1840 (972–5254)	2156 (788–3852)	0.885	U = 94
APRI, median (range)	2.30 (0.1–18.74)	6.56 (0.31–18.88)	0.201	U = 59
Fib-4, median (range)	2.47 (0.28–10.92)	5.24 (1.47–11.62)	0.078	U = 48

Anti-HBs positivity was observed in one patient (4.3%) in the anti-HBc IgG-negative group and 11 patients (84.6%) in the anti-HBc IgG-positive group (p<0.001). When comparing AIH patients by anti-HBc IgG status, total bilirubin at diagnosis was significantly higher in the anti-HBc IgG-positive group. However, liver biopsy findings-including Knodel HAI and Ishak grade-did not differ significantly between groups.

After one year of treatment, the anti-HBc IgG-positive group showed higher AST, direct bilirubin, and Fib-4 scores, along with lower albumin levels compared to the negative group. Additionally, more patients in this group had AST >40 U/L, total bilirubin >1.2 mg/dL, direct bilirubin >0.3 mg/dL, albumin <40 g/L, and Fib-4 scores <1.45 or >3.25 (Table 2).

**Table 2:** Comparison between anti-HBc IgG-negative and positive groups in patients with autoimmune hepatitis P-values were calculated using the Mann-Whitney U test or independent samples t-test for continuous variables, and the Chi-square test for categorical variables. Statistical significance was set at p<0.05 ALT: alanine aminotransferase; APRI: AST to platelet ratio index; AST: aspartate aminotransferas; Fib-4: Fibrosis-4; GGT: gamma-glutamyl transferase; IgG: immunoglobulin G; INR: international normalized ratio; SD: standard deviation

Variable	Anti-HBc IgG (-) n=23	Anti-HBc IgG (+) n=13	P-value	Test statistic
Gender, n (%)	Female 19 (82.6%)	Female 12 (92.3%)	0.419	χ² = 0.653
	Male 4 (17.4%)	Male 1 (7.7%)		
Age, years, median (range)	48 (18–59)	52 (41–65)	0.04	U = 211.5
Knodel HAI, median (range)	9/18 (3/18–13/18)	7/18 (3/18–12/18)	0.448	U = 126
Ishak score, median (range)	3/6 (0–6/6)	4/6 (0–5/6)	0.791	U = 139.5
ALT, U/L, median (range)	233 (7–2020)	422 (28–1055)	0.558	U = 168
ALT first year, U/L, median (range)	20 (8–44)	30 (6–547)	0.107	U = 199
Patients with ALT >40 U/L in first year, n (%)	5 (21.7%)	5 (36.1%)	—	—
AST, U/L, median (range)	143 (16–1357)	436 (24–1224)	0.379	U = 177
AST first year, U/L, median (range)	21 (13–40)	27 (9–501)	0.047	U = 209.5
AST/ALT ratio, mean ± SD	0.90 ± 0.34	1.09 ± 0.10	0.107	t(34) = -1.58
AST/ALT ratio first year, median (range)	1.07 (0.72–2.63)	1.2 (0.69–4.01)	0.673	U = 163
Patients with AST >40 U/L in first year	0	5 (36.1%)	—	—
Total bilirubin, mg/dL, median (range)	1.85 (0.2–10.6)	8.3 (0.5–19.7)	0.041	U = 202
Total bilirubin first year, mg/dL, median (range)	0.45 (0.2–1.3)	0.5 (0.3–30)	0.312	U = 180.5
Patients with total bilirubin >1.2 mg/dL in the first year	0	3 (23.1%)	—	—
Direct bilirubin, mg/dL, median (range)	0.7 (0.1–7.2)	6.1 (0.1–15.5)	0.07	U = 204.5
Direct bilirubin first year, mg/dL, median (range)	0.2 (0.1–0.4)	3.7 (0.2–25)	0.02	U = 220
GGT, U/L, median (range)	153 (7–822)	206 (37–831)	0.344	U = 178.5
GGT first year, U/L, median (range)	34 (7–266)	47 (10–450)	0.267	U = 183.5
Patients with direct bilirubin >0.3 (mg/dL) first year, n (%)	3 (13%)	5 (36.1%)	—	—
INR, median (range)	1.11 (0.9–1.93)	1.14 (1.01–1.78)	0.159	U = 193
INR first year, median (range)	1.03 (0.9–1.28)	1 (0.92–4.59)	0.697	U = 161.5
Albumin, g/L, mean ± SD	40.32 ± 1.16	37 ± 1.56	0.093	t(34) = 1.82
Albumin first year, g/L, mean ± SD	43.8 ± 3.41	39.1 ± 1.88	0.043	t(34) = 2.69
Patients with albumin <40 g/L, n (%)	9 (39.1%)	9 (69.2%)	—	—
Patients with Albumin <40 g/L in the first year, n (%)	1 (4.3%)	5 (36.1%)	—	—
Fib-4 score, median (range)	2.11 (0.28–10.92)	3.99 (0.75–11.62)	0.065	U = 206
Fib-4 score first year, median (range)	0.69 (0.18–1.95)	1.37 (0.54–5.13)	0.008	U = 229
Patients with Fib-4 score <1.45, n (%)	8 (34.8%)	2 (15.4%)	—	—
Patients with Fib-4 score <1.45 in the first year, n (%)	16 (69.6%)	8 (61.5%)	—	—
Patients with Fib-4 score <1.45 in thefirst year among patients with Fib-4 score >1.45 at baseline, n (%)	8/15 (53.3%)	6/11 (54.5%)	—	—
Patients with Fib-4 score <3.25, n (%)	16 (69.6%)	4 (30.8%)	—	—
Patients with Fib-4 score <3.25 in the first year, n (%)	23 (100%)	10 (76.9%)	—	—
IgG, mg/dL, median (range)	1771 (972–5254)	2554 (788–4251)	0.19	U = 190
APRI, median (range)	1.7 (0.1–18.74)	3.48 (0.2–18.88)	0.515	U = 170
APRI first year, median (range)	0.23 (0.1–0.48)	1.19 (0.16–12.78)	0.087	U = 202

**Figure 1:**
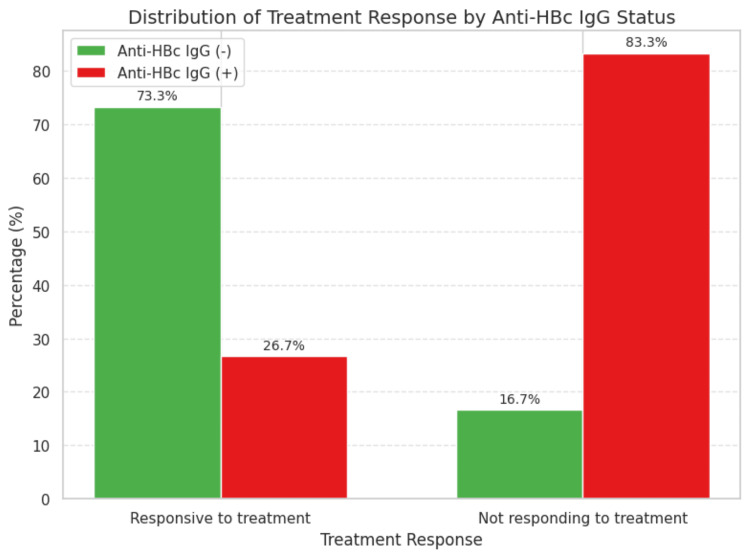
Treatment response among patients by anti-HBc IgG status The difference between the two groups was statistically significant (p = 0.008) IgG: immunoglobulin G

Among patients negative for anti-HBc IgG, 22 out of 23 (73.3%) responded to treatment, compared to only eight out of 13 (26.7%) patients in the anti-HBc IgG-positive group. This difference was statistically significant (p = 0.008) (Figure 1).

**Figure 2:**
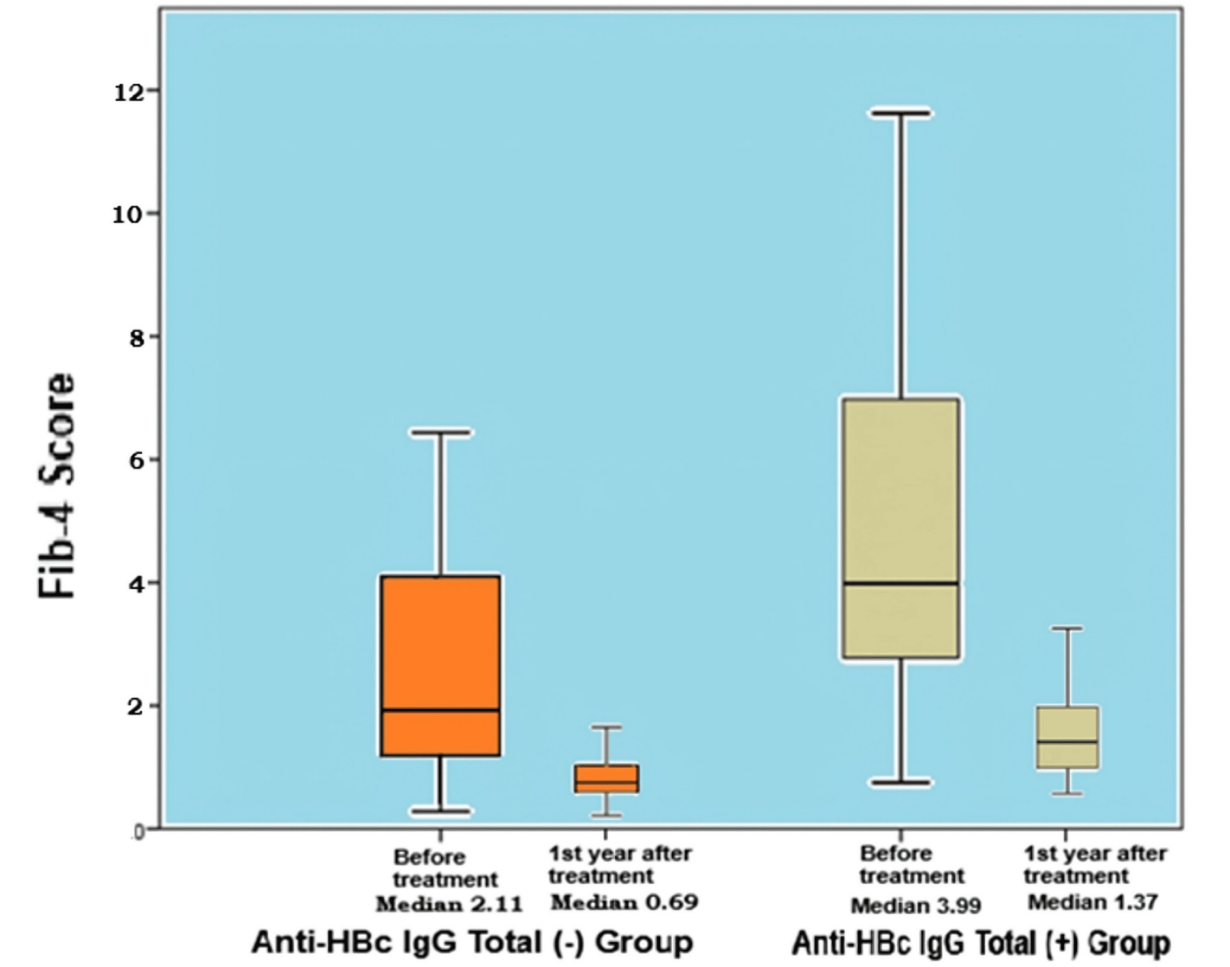
Change in Fib-4 score before and after one year of treatment in anti-HBc IgG-negative and positive patients (illustrated using box plots) In the anti-HBc IgG-negative group, there was a significant decrease in Fib-4 scores, with a median of 2.11 before treatment and 0.69 after one year (p<0.001), indicating a marked improvement in liver fibrosis. On the other hand, anti-HBc IgG-positive patients experienced a moderate decrease in their Fib-4 scores, with a median of 3.99 before treatment and 1.37 after one year (p = 0.002). Though still showing improvement, the reduction in Fib-4 scores in this group was less pronounced compared to the anti-HBc IgG-negative group Fib-4: Fibrosis-4; IgG: immunoglobulin G

When comparing baseline values to those at one year post-treatment in the anti-HBc IgG-negative group, significant improvements were observed in liver function tests, the APRI index, and the Fib-4 score (Table 3, Figure 2).

**Table 3:** Comparison of the anti-HBc IgG (-) group between the beginning and the first year after treatment ALT: alanine aminotransferase; APRI: AST to platelet ratio index; AST: aspartate aminotransferas; Fib-4: Fibrosis-4; GGT: gamma-glutamyl transferase; IgG: immunoglobulin G; INR: international normalized ratio; SD: standard deviation

Variables	Before treatment	First year after treatment	P-value	Test statistic
ALT, U/L, median (range)	233 (7-2020)	20 (8-44)	<0.001	Wilcoxon Z = -4.167
AST, U/L, median (range)	143 (16-1357)	21 (13-40)	<0.001	Wilcoxon Z = -4.197
AST/ALT ratio, median (range)	0.84 (0.44-1.76)	1.07 (0.72-2.63)	0.002	Wilcoxon Z = -3.072
Total bilirubin, mg/dL, median (range)	1.85 (0.2-10.6)	0.45 (0.2-1.3)	<0.001	Wilcoxon Z = -3.596
Direct bilirubin, mg/dL, median (range)	0.7 (0.1-7.2)	0.2 (0.1-0.4)	<0.001	Wilcoxon Z = -3.555
GGT, U/L, median (range)	153 (7-822)	34 (7-266)	<0.001	Wilcoxon Z = -3.912
INR, median (range)	1.1 (0.9-1.93)	1.02 (0.9-1.28)	0.026	Wilcoxon Z = -2.226
Albumin, g/L, mean ± SD	40.32 ± 1.16	43.8 ± 3.41	0.017	t = -2.58
APRI, median (range)	1.7 (0.1-18.74)	0.23 (0.1-0.48)	<0.001	Wilcoxon Z = -4.167
Fib-4, median (range)	2.11 (0.28-10.92)	0.69 (0.18-1.95)	<0.001	Wilcoxon Z = -4.067

When comparing baseline values to those at one year post-treatment in the anti-HBc IgG-positive group, no significant improvement was observed in total bilirubin, direct bilirubin, INR, or albumin levels (Table 4).

**Table 4:** Comparison of the anti-HBc IgG (+) group between the beginning and the first year after treatment ALT: alanine aminotransferase; APRI: AST to platelet ratio index; AST: aspartate aminotransferas; Fib-4: Fibrosis-4; GGT: gamma-glutamyl transferase; IgG: immunoglobulin G; INR: international normalized ratio; SD: standard deviation

Variables	Before treatment	First year after treatment	P-value	Test statistic
ALT, U/L, median (range)	422 (28-1055)	30 (6-547)	0.002	Wilcoxon Z = -3.11
AST, U/L, median (range)	436 (24-1224)	27 (9-501)	0.002	Wilcoxon Z = -3.059
AST/ALT ratio, median (range)	1.01 (0.52-1.66)	1.2 (0.69-4.01)	0.345	Wilcoxon Z = -0.943
Total bilirubin, mg/dL, median (range)	8.3 (0.5-19.7)	0.5 (0.3-30)	0.415	Wilcoxon Z = -0.934
Direct bilirubin, mg/dL, median (range)	6.1 (0.1-15.5)	3.7 (0.2-25)	0.286	Wilcoxon Z = -1.067
GGT, U/L, median (range)	206 (37-831)	47 (10-450)	0.009	Wilcoxon Z = -2.621
INR, median (range)	1.14 (1.01-1.78)	1 (0.92-4.59)	0.422	Wilcoxon Z = -0.804
Albumin, g/L, mean ± SD	37 ± 1.56	39 ± 1.88	0.215	t = -1.31
APRI, median (range)	3.48 (0.2-18.9)	1.19 (0.1-12.8)	0.004	Wilcoxon Z = -3.059
Fib-4, median (range)	3.99 (0.75-11.62)	1.37 (0.54-5.13)	0.002	Wilcoxon Z = -2.9

Fib-4 scores in anti-HBc IgG-positive patients demonstrated a moderate decrease after one year of treatment (median before: 3.99; after: 1.37; p = 0.002). Although this represents improvement, the reduction was less pronounced compared to that observed in anti-HBc IgG-negative patients (Figure 2).

## Discussion

Latent hepatitis B and impaired treatment response in autoimmune hepatitis

In our study, patients with latent hepatitis B infection, as indicated by anti-HBc IgG positivity, demonstrated significantly worse biochemical outcomes and reduced treatment response compared to those without prior HBV exposure. Total bilirubin, AST, and direct bilirubin levels were significantly elevated at diagnosis and remained persistently high after one year of standard immunosuppressive therapy. Moreover, the percentage of patients achieving normalization of liver enzymes and synthetic function parameters (such as albumin) was markedly lower in the Anti-HBc IgG positive group, suggesting that latent HBV may interfere with therapeutic success in AIH. These findings align with previous reports that have identified a higher prevalence of occult or latent hepatitis B infection in patients with autoimmune liver diseases [1-4]. Studies by Georgiadou et al. and Chen et al. have demonstrated the presence of intrahepatic HBV-DNA in AIH patients despite being HBsAg-negative, highlighting the concept of occult HBV infection and its potential to contribute to persistent hepatic injury [5,6]. Batskikh et al. further suggested that a history of HBV infection could play a role in disease pathogenesis by modulating immune tolerance mechanisms [4].

Pathophysiological considerations

Latent HBV infection may contribute to chronic hepatic inflammation through low-grade antigenic stimulation or subclinical viral replication. Immunosuppressive therapies such as corticosteroids and azathioprine, which are first-line treatments in AIH, may further impair antiviral immune responses and allow for viral reactivation or ongoing antigen expression, even in the absence of detectable HBV-DNA in serum [7,8]. Additionally, the hepatitis B virus has been shown to modulate T-cell responses and impair antigen presentation, possibly reducing the effectiveness of immunosuppressive therapy in controlling autoimmune-mediated liver injury [9]. This ongoing hepatic insult may explain the persistently elevated liver enzymes and incomplete normalization of Fib-4 scores observed in anti-HBc-positive patients.

Clinical implications and screening recommendations

Despite these potential risks, routine screening for latent HBV infection is not yet a standard part of AIH management protocols. Most guidelines, such as those from the American Gastroenterological Association (AGA) and European Association for the Study of the Liver (EASL), recommend HBV screening only for patients at high risk of reactivation (e.g., those receiving B-cell depleting agents or undergoing chemotherapy) [10-13]. However, our findings, consistent with others in the literature, suggest that even standard immunosuppressive regimens used in AIH may be compromised by latent HBV infection [4-6,8].

In our cohort, anti-HBc IgG positivity was associated with significantly lower treatment response rates (26.7% vs. 73.3%, p = 0.008), higher baseline bilirubin levels, and less pronounced reductions in Fib-4 scores. These findings support broader HBV screening strategies in AIH, including tests for anti-HBc IgG, HBsAg, anti-HBs, and, where relevant, HBV-DNA-even in the absence of immunosuppressive regimens known for high reactivation risk [10-12].

For anti-HBc-positive patients-particularly those without protective anti-HBs or with detectable HBV-DNA-antiviral prophylaxis with agents such as entecavir or tenofovir could be considered as a preventive strategy to reduce hepatic inflammation and improve response to AIH therapy [11,12]. Studies have shown that such prophylaxis is safe and effective in similar immunosuppressive contexts [13,14].

Limitations and future research directions

This study has several limitations, most notably its retrospective design and relatively small sample size. Furthermore, HBV-DNA was not tested in all anti-HBc-positive patients, limiting our ability to distinguish between resolved and occult infection. The absence of detailed treatment protocols is another limitation, as the variability in treatment regimens across patients could introduce bias. Additionally, the study was conducted at a single center, which may limit the generalizability of its findings to broader populations. Despite these limitations, our data provide important real-world insights and support the hypothesis that latent HBV infection may adversely affect AIH treatment outcomes. Future prospective studies are needed to confirm the association between anti-HBc positivity and treatment resistance in AIH, evaluate the role of antiviral prophylaxis in improving treatment response, and explore immunopathological mechanisms underlying the interaction between latent HBV and autoimmunity.

Clinical recommendations

Based on the findings of our study and supporting evidence from the literature, we propose that all patients newly diagnosed with AIH be screened for anti-HBc IgG, HBsAg, and anti-HBs, with HBV-DNA testing in anti-HBc-positive individuals. Prophylactic antiviral therapy may be considered in anti-HBc-positive patients, particularly those who are anti-HBs-negative or have detectable HBV-DNA, although further research is needed to assess its impact on clinical outcomes in this group. Liver function tests and Fib-4/APRI scores should be monitored closely throughout treatment, and antiviral therapy should be considered early in patients who demonstrate a poor biochemical response. HBV testing should be incorporated into the initial evaluation and long-term follow-up of AIH patients, especially when standard therapy fails to produce an adequate response.

## Conclusions

Latent hepatitis B infection, as evidenced by anti-HBc IgG positivity, appears to negatively influence treatment response and liver function recovery in patients with AIH. Our findings underscore the importance of systematic HBV screening and the potential role of antiviral prophylaxis in this subgroup. While latent HBV may be associated with poorer treatment outcomes in AIH, the potential role of prophylactic antivirals remains speculative. Further prospective studies are needed to evaluate the impact of antiviral therapy in this subgroup before any clinical recommendations can be made.
